# Effect of chronic administration of ostruthin on depression-like behavior in chronically stressed mice

**DOI:** 10.1016/j.ibneur.2024.05.009

**Published:** 2024-05-22

**Authors:** Masayoshi Okada, Thi Thu Thuy Tran

**Affiliations:** a Department of Medical LifeScience, College of Life Science, Kurashiki University of Science and the Arts, Kurashiki, Okayama 712-8505, Japan; b Institute of Natural Products Chemistry, Vietnam Academy of Science and Technology, Hanoi, Viet Nam

**Keywords:** Ostruthin, Antidepressant, Chronic unpredictable mild stress, TREK-1 channel, And drug-metabolizing enzyme

## Abstract

We have previously shown that a single dose of a TREK-1 channel activator, ostruthin, exhibited antidepressant and anxiolytic effects in acute behavioral test models in mice. To assess the potential clinical application, it is essential to evaluate the effects of long-term administration of ostruthin in a chronically stressed mouse model, which is considered to be similar to the clinical condition of major depression in humans. Here, we tested the effects of a single and a 7-day administration of ostruthin on mice that were subjected to chronic unpredictable mild stress (CUMS). A single administration of ostruthin showed antidepressive effects in the tail suspension and forced swim tests of CUMS-treated mice. Unexpectedly, the 7-day administration exhibited only insignificant antidepressive and anxiolytic effects. The 7-day regimen did not affect food intake or body-weight gain, suggesting the absence of apparent cytotoxicity. The mice receiving the 7-day administration had significantly lower blood concentrations of ostruthin compared to those receiving a single dose, suggesting an upregulation of drug-metabolizing activities. These findings suggest that there is a need for stable TREK-1 channel activators that are not affected by drug metabolism.

## Highlights

• Previously, ostruthin, a TREK-1 channel activator, showed an antidepressant effect.
• But, the effect on chronically stressed mice has not been evaluated.
• A single shot of ostruthin had antidepressant effects on chronically stressed mice.
• But, a 7-day administration did not, likely due to drug-metabolizing activity.
• These results suggest a need for stable TREK-1 channel activators.

## 1 Introduction

Depression has a major impact on personal lives, with an annual incidence rate of 7.1% among adults in the United States, resulting in enormous societal losses (Zhdanava et al., 2021). This disorder is commonly treated with antidepressants, such as selective serotonin reuptake inhibitors (SSRIs), serotonin-norepinephrine reuptake inhibitors (SNRIs), noradrenergic and specific serotonergic antidepressants (NaSSAs), and dopamine system stabilizers (DSS), which modulate the transmission of these monoamines. However, according to the STAR*D study, conducted by the American National Institute of Mental Health, 30% of depressed patients do not respond to current antidepressants (Dibernardo et al., 2018). This highlights the need for antidepressants with novel mechanisms of action.

It has been suggested that patients with depression may have dysregulated neuronal activity in certain brain nuclei. A recent study has reported a correlation between the severity of depression symptoms and the changes in neuronal activity in some nuclei (Xiao et al., 2023). Indeed, neuromodulation techniques, such as deep brain stimulation, have shown therapeutic effects in patients with intractable depression. These stimulations are thought to re-regulate the dysregulated neuronal activity (Mitra et al., 2023). Therefore, it is expected that other neuromodulatory methods will also achieve therapeutic effects.

Neuronal intrinsic excitability is regulated mainly by K⁺ channel currents. There are 76 types of channels in the channel families (Kuang et al., 2015), each expressed in a nucleus-specific pattern in different neuronal nuclei (Jenkinson, 2006). Modulations of these channels can therefore modulate neuronal activities in a nucleus-specific manner and thereby have therapeutic effects on depression (Joseph et al., 2018, Okada et al., 2020, Okada and Ortiz, 2022). Indeed, previous research has demonstrated that the activation or inhibition of K⁺ channels can have antidepressant effects on mouse models of depression by modulating neural activity in specific nuclei. For example, an activator for a KCNQ-type K⁺ channel was found to have an antidepressant effect by normalizing the hyperactivity of dopaminergic neurons in the ventral tegmental area (Friedman et al., 2016). The TWIK-related K⁺ channel, TREK-1, is a member of the two-pore domain K⁺ (K2P) channels that are responsible for background K⁺ currents. Knock-out mice lacking TREK-1 exhibited an antidepressant phenotype (Heurteaux et al., 2006), and a TREK-1 channel blocker also showed antidepressive effects (Djillani et al., 2017). The authors suggested that the manifestation of the antidepressive effect was due to the blockade of that channel in the dorsal raphe nucleus. Interestingly, we have shown that an activator for the TREK-1 channel, ostruthin, has antidepressant and anxiolytic effects (Joseph et al., 2018). That study found that these effects involve the suppression of neural activities in a different nucleus, the lateral septum. In addition, we also demonstrated that the suppression of this nucleus is also involved in the antidepressive effect of ROMK (Kir1.1) channel blocker, tertiapin-RQ (Okada et al., 2020). Thus, K⁺ channel modulators, e.g., ostruthin, are promising as antidepressants and should be further investigated for clinical application.

However, the effects of long-term administration of ostruthin in a chronic unpredictable mild stress (CUMS) model of depression, conditions which mimic human depression in several aspects (Antoniuk et al., 2019), have not been tested. Meanwhile, ostruthin has been reported to have cytotoxic effects on smooth muscle and cancer cells (Joa et al., 2011, Nguyen et al., 2017), therefore the possible adverse effects of long-term administration of ostruthin needs to be evaluated. In this report, we examined the effects of ostruthin administration for 1 and 7 days on several behavioral tests of depression- and anxiety-like behavior on mice that were subjected to the CUMS.

## 2 Materials and methods

### 2.1 Purification of ostruthin

The roots of *Paramignya trimera* were collected in Khanh Hoa province Vietnam in April 2014. The plant material was taxnomically identified by Dr. Nguyen Quoc Binh (Vietnam National Museum of Nature, Hanoi, Vietnam). A voucher specimen (PT042021) has been deposited at the Institute of National Products Chemistry, Vietnam Academy of Science and Technology, Hanoi, Vietnam. The dried material (200 g) was powdered and extracted with methanol at room temperature, and the methanol was evaporated under reduced pressure at 45℃. The crude extract was dissolved in CH_2_Cl_2_ at room temperature with sonication. After evaporating the solvent at 40℃, the sample was separated into seven fractions by silica gel column chromatography. The fourth fraction was again chromatographed on a silica gel column with an increasing concentration of ethyl acetate mixed with n-hexane (5.0 ± 6.7%). Ostruthin was purified to homogeneity (> 99.1%) according to the chromatogram of A_330 nm_.

### 2.2 Experimental animals

Male ICR mice (n = 80 in total) aged 5 weeks were purchased from Shimizu Laboratory Supplies (Kyoto, Japan). They had free access to food and water and were kept in a 12-h light/dark cycle (08:00–20:00). The mice were maintained for a week before experiments. The animals were kept in groups in plastic cages except for the last week of CUMS, in which they were individually reared in small cages. Experiments were conducted in a quiet and air-conditioned room between 10:00 and 18:00. Animal experiments were carried out following the guidelines of the Physiological Society of Japan and were approved by the Committee on Animal Experiments at Kurashiki University of Science and the Arts.

After one week of adaptation, mice were subjected to CUMS for six weeks and underwent behavioral tests according to the previous methods (Wen et al., 2019). CUMS procedure was performed as illustrated in Table 1. Mice were subjected to eight kinds of stressors in a pseudo-random and unpredictable manner for six weeks, with one stressor in the morning (9:00 – 13:00) and another in the afternoon (15:00 – 20:00) at irregular timings. A representative schedule is depicted in Table 1. Each stressor was administered in approximately equal numbers, except for the inverted light and dark cycles on weekends. The stressors included: swimming in a 2 L beaker of water at a temperature of 25–27℃ for 10 min, constriction in a breathable tube for 1 hour, food and water deprivation for 14 hours, exposure to wet bedding (250 mL water and soggy sawdust) for 3 hours, shaking of the cage (reciprocal movement of 3–4 cm at approximately 120 rpm) for 1 hour, tilting of the cage at a 45° angle for 1 hour, exposure to an inverted light and dark cycle (from 8: 00–20:00–20:00–8:00) for 48 hours, and exposure to strobe light (120 flashes/min) for 3 hours.

**Table 1:** Typical weekly schedule of stress loading.

	Mon	Tue	Wed	Thu	Fri	Sat	Sun
AM	Tilted	Forced	Cage	Tilted	Constriction	Inverted	Inverted
	cage	swim	shaking	cage		L/D cycle	L/D cycle
PM	Constriction	Strobe	Wet	F & W	Cage	Inverted	Inverted
		light	bedding	deprivation	shaking	L/D cycle	L/D cycle

CUMS and behavioral tests on mice were conducted in five rounds of experiments (Table 2). The first round was conducted for the confirmation of the induction of depression-like behavior with CUMS treatment, without drug administration. CUMS mice were chronically stressed as described above, whereas control mice were daily weighed and kept in cages enriched with cardboard tubes (n = 12 and 10). The second and third rounds involved a single dosing of ostruthin and behavioral testing, whereas the fourth and fifth rounds involved a 7-day dosing. Each round of experiments was performed with 6 animals for both PBS and ostruthin (n = 48, total). The results of depressive-like behavior of the 7-day dosing were analyzed together with the results of 4th and 5th rounds. In the 6th round, mice were used for the measurement of blood ostruthin concentration without being subjected to CUMS (n = 5 and 5).

**Table 2:** Schedule of behavioral tests.

Experiment Round (# of mice) (male)				
	Administration	Behavioral and other tests		
		The day before -------	24 hrs	------- Last day
1st round (n =22)	N/A	NSF, FST	SPT	TST, AWT
2nd round (n = 12)	Ostruthin or PBS Single (the last day)			NSF, TST, AWT
3rd round (n = 12)	Ostruthin or PBS Single (the day before)	FST	SPT	
4th round (n = 12)	Ostruthin or PBS 7 days	NSF, FST, EPM	SPT, food intake	TST, AWT OFT, LDT
5th round (n = 12)	Ostruthin or PBS 7 days	NSF, FST	SPT, food intake	TST, AWT
6th round (n = 10)	Ostruthin Single or 7 days			Blood collection

Ostruthin was initially dissolved in DMSO at a higher concentration (100 mM) and then diluted in phosphate-buffered saline (PBS) to a final concentration of 1.67 mM. The diluted solution was mixed vigorously for more than 10 min before the experiments. Ostruthin was administered intraperitoneally (i.p.) at a dose of 5 mg/kg (0.01 mL/g) body weight). This dose was chosen based on our previous study, which showed a bell-shaped dose-response relationship for the anxiolytic effect, with the highest effect observed at 5 mg/kg (Joseph et al., 2018). In the 7-day dosing mice, ostruthin was administrated between two stress procedures in the last week. The control mice were injected intraperitoneally with an equal volume of PBS containing 1/60 vol of DMSO.

#### 2.2.1 Behavioral tests

Depression-like behaviors were tested with five behavioral tests, which included the tail-suspension test (TST), forced swim test (FST), activity wheel test (AWT), sucrose preference test (SPT), and novelty-suppressed feeding (NSF). Anxiety-like behaviors were tested with elevated plus maze (EPM), open-field test (OFT), and light/dark box test (LDT). These tests were carried out 30–60 min after the injection of ostruthin or PBS with 5 – 30 min intervals between tests as shown in the typical schedule (Table 2). Mice from the first to fifth rounds were subjected to 2–8 different tests on the last day and the day before (see Table 2). For the NSF test, food and water were deprived from 20:00 on the day before, until the end of the NSF test. In the fourth and fifth rounds, both depression- and anxiety-like behaviors were tested on the 6th and 7th days of the administration. After tests on the 6th day, mice were subjected to SPT for 24 hrs, and sucrose solution/water consumption and food intake were measured. Each behavioral test was performed according to a method reported previously (Joseph et al., 2018, Okada et al., 2020)

#### 2.2.2 Forced swim test (FST)

Briefly, a mouse was placed into a 2 L beaker containing 12 cm of water (25–27℃). The mouse was allowed to swim for 6 min. The last 4 min of data were used for analysis. Immobility is defined as the absence of activity, such as escape-oriented behaviors. The slow movement of the forelimbs only for breathing was considered immobility.

#### 2.2.3 Tail suspension test (TST)

The mouse was hung on a hook using adhesive tape placed 1 cm from the extremity of its tail 50 cm above the table. The amount of time spent immobile was recorded for 5 min.

#### 2.2.4 Activity Wheel test (AWT)

Five min after the TST, the mouse was subjected to the voluntary wheel running test. The number of rotations was measured for 5 min using an activity monitoring running wheel (Muromachi Kikai, Tokyo, Japan).

#### 2.2.5 Novelty-suppressed feeding (NSF)

After the preceding food and water deprivation overnight, the test mouse was placed in a corner of the arena and allowed to explore the open field (90 × 90 × 45 cm). Food pellets were placed on filter paper (10 cm in diameter) in the center of the arena. The time it took for the mouse to approach and take the first bite of chow was measured. If the mouse did not feed, the trial was terminated after 5 min (results shown in Fig. 2, Fig. 3 min (Fig. 3).

#### 2.2.6 Sucrose preference test (SPT)

Mice were initially acclimated to sucrose solution on the day before dosing and on the first day of the 7-day dosing period. Acclimation consisted of the free choice of drinking either 1% (w/v) sucrose solution or tap water. Food and water were withheld from 20:00 h on day 5 until the drug administration on day 6. Consumption of sucrose solution and water for 24 h was then estimated by measuring the difference in weight of the respective bottles before and after. The percentage of sucrose preference was calculated according to the following formula: sucrose preference (%) = sucrose intake / (sucrose intake + water intake) * 100.

#### 2.2.7 Open-field test (OFT)

The open field consisted of a square (90 × 90 × 45 cm), in which the floor was divided into 16 equal squares. After a mouse was placed in a corner of the open field facing the wall, the number of line crossings of the four central squares, and total line crossings were counted for 5 min. After the removal of the animal, the apparatus was cleaned and wiped.

#### 2.2.8 Elevated plus maze (EPM)

The elevated plus maze apparatus consisted of four arms set in a cross pattern from a neutral central square (6 × 6 cm). Two opposite arms were delimited by vertical walls (closed arms, 30 × 15 × 6 cm), whereas the other two arms had unprotected edges (open arms, 30 × 6 cm). The maze was elevated 40 cm above the floor. At the beginning of each 5 min test session, a mouse was placed in the central square. The total number of entries to open arms and time spent in the open arms were measured. After the removal of the animal, the apparatus was cleaned and wiped.

#### 2.2.9 Light/Dark box test (LDT)

The light/dark box consisted of two compartments with a total exterior size of 46 × 27 × 30 cm. One-third of the box was used as a dark compartment (< 5 Lux), which was covered with a black wall and lid, and the rest of the area was used as a light compartment (400 Lux). These compartments were connected via a small opening (7 × 7 cm), enabling a transition between the two compartments. The mouse was placed in a corner of the light area and the time spent in the light area and the number of transitions between the light and dark compartments was measured for 5 min.

### 2.3 HPLC analysis of ostruthin concentration in blood

To measure blood ostruthin concentration, we collected the blood from the inferior vena cava of the mice 30 min after the last administration. Serum samples were collected by centrifugation at 1,500 × g for 15 min at 4℃ after clotting for 30 min on ice. For deproteination, we added 200 μL of acetonitrile to each serum sample (50 μL) and incubated the samples for 15 min on ice. The samples were centrifuged at 1,500 × g for 15 min at 4℃, and the supernatant was collected. The supernatant (20 μL) was analyzed by high-performance liquid chromatography (HPLC) using a reversed-phase column (Crestpak C18S, JASCO, Tokyo, Japan). Ostruthin was eluted with a gradient from 0.1% formic acid in H_2_O to 100% acetonitrile, monitoring A_333 nm_ with a UV detector (JASCO). The concentration was estimated from the peak height at a retention time of 22.2 min.

### 2.4 Statistical analysis

Data are given as the mean ± SEM. The normality of data distribution was tested with the Shapiro-Wilk test. If data were normally distributed, statistical significance between the two groups was determined using Student's t-test. If not, data were analyzed with the Mann-Whiteny U-test. A p-value of <0.05 was considered significant. The number of asterisks indicates the p values: *, p<0.05; **, p < 0.01; ***, p<0.005.

## 3 Results

### 3.1 Antidepressive effects of single administration of ostruthin on CUMS-treated mice

CUMS treatment was expected to induce depression-like behaviors in these tests (Antoniuk et al., 2019). We first compared the depression-like behavior in FST and TST of CUMS-treated mice with that of control (unstressed) mice, before examining the effect of ostruthin. The treatment significantly increased the immobile time in FST (38.8 ± 10.6 and 130.2 ± 14.0 sec, p < 0.0005, Student’s t-test, n = 10 and 12) and insignificantly increased that in the TST (43.8 ± 9.6 and 66.5 ± 17.9 sec, p = 0.201). These results suggest that the CUMS-treatment had the expected effect on the mice's behavior.

**Fig. 1:**
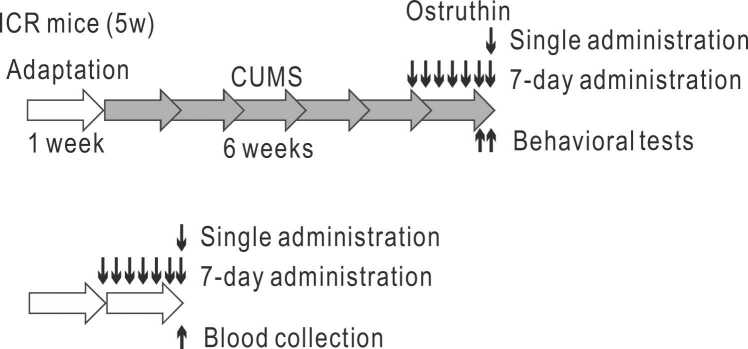
Experimental design of CUMS, drug administration, and behavioral tests.

**Fig. 2:**
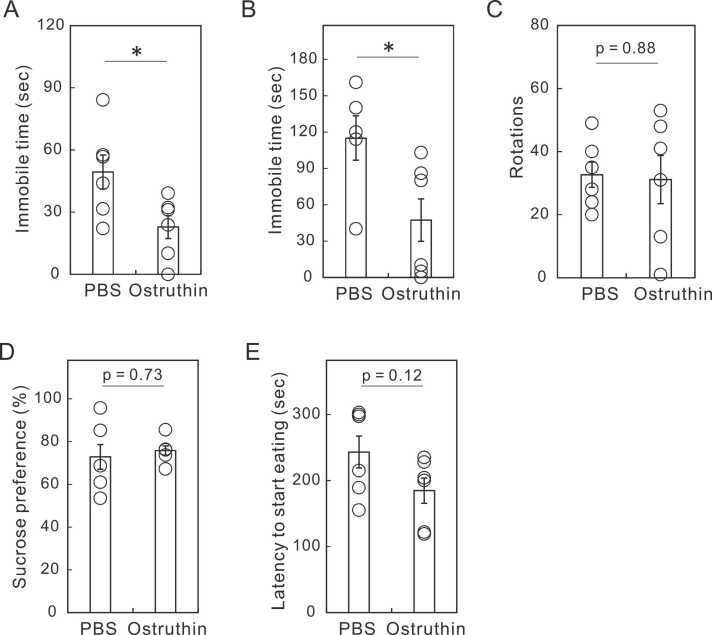
Antidepressive effects of single administration of ostruthin on CUMS-treated mice. (A) Shortening of immobile time at TST. (B) Shortened immobile time at FST. (C) Lack of effect on the spontaneous motor activity at AWT. (D) Little antidepressive effect at SPT. (E) Shortening tendency of latency to start eating at the NSF. The results suggest that the antidepressant effects are of varying degrees. These behaviors were conducted 30–60 min after the single administration of ostruthin. (n = 6).

To investigate the effects of single dosing of ostruthin, we intraperitoneally administered ostruthin, or an equal volume of PBS, to the ICR mice treated with CUMS for 6 weeks (Fig. 1) and examined its effects on depression-like behavior 30 min after administration (Table 2 and Fig. 2). The dose administrated was 5 mg/kg, which was previously determined to have the highest anxiolytic effect (Joseph et al., 2018). As expected, the single administration of ostruthin decreased the immobile time in both FST and TST, indicating its antidepressive effects on CUMS-treated mice (Fig. 2A and B). We also tested the effects in AWT, SPT, and NSF: a single administration of ostruthin showed insignificant antidepressive effects (Fig. 2C-E).

### 3.2 Diminishment of antidepressive effects of 7-day administration of ostruthin

**Fig. 3:**
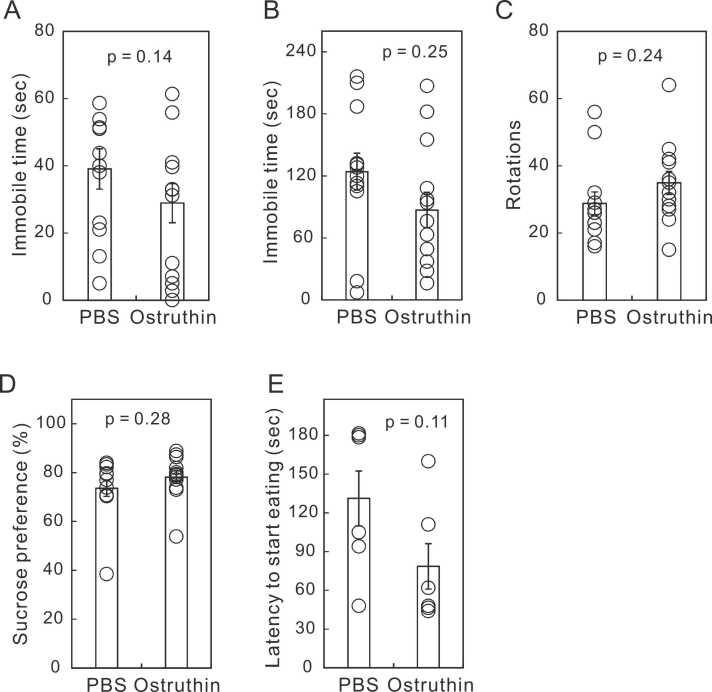
Diminishment of antidepressive effect after 7 days of ostruthin administration. (A) Immobile times in TST. (B) Immobile times in FST. (C) Spontaneous locomotor activity in AWT. (D) Sucrose preference in SPT. (E) Latency to start eating in NSF. All of these results indicated a trend toward antidepressant effects, but none were statistically significant. (n = 12).

**Fig. 4:**
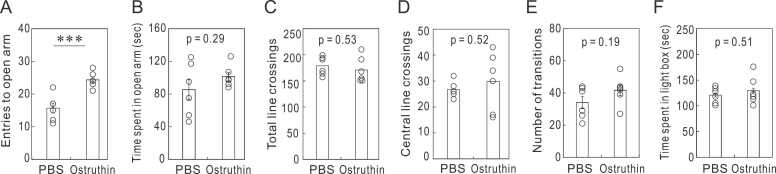
Diminishment of anxiolytic effect in the 7-day regimen mice. The 7-day administration of ostruthin showed a significant anxiolytic effect in the number of entries to the open arm in EPM (A). However, the effect was insignificant in the time spent in the open arm (B). There were no substantial differences in the total and central line crossings OFT (C and D) the number of transitions and the time spent in the lightbox in LDT (E and F). n = 6.

Considering that depression is a long-term condition and antidepressants are taken over a long period, we examined the effects of the 7-day administration of ostruthin on depression-like behavior. Unexpectedly, the 7-day administration only showed insignificant antidepressive effects in TST and FST (Fig. 3A and B). Similarly, the mice treated for 7 days showed antidepressive tendency in AWT, SPT and NSF (Fig. 3C, D, and E). We also tested anxiety-like behavior after the 7-day administration with OFT, LDT, and EPM (Fig. 4). The one-week administration showed the anxiolytic effect only at the entry to the open arm in EPM (Fig. 4A), with no effects observed in the other tests (Fig. 4B-F).

### 3.3 The decreased blood concentration of ostruthin after 7-day administration

**Fig. 5:**
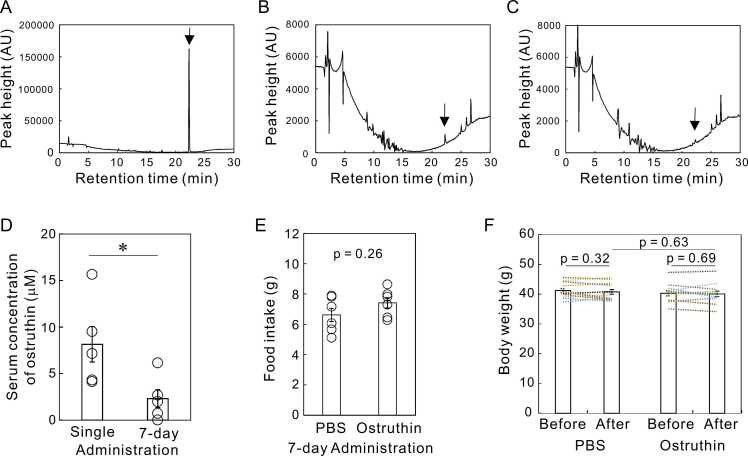
Reduced blood ostruthin concentration and lack of effect on food intake and body weight. (A)　Chromatograms of purified ostruthin. Ostruthin diluted in PBS (concentration 1.67 mM) was fractionated by a C-18 reversed-phase column. The ordinate indicates the A_333 nm_ (arbitral unit of UV-detector). A peak was found with a retention time of 22.2 min (arrow). (B) Chromatogram of the deproteinized mouse serum collected 30 min after single i.p. administration of ostruthin. (C) Chromatogram of serum sample after 7-day administration. (D) Estimated serum ostruthin concentrations after single and 7-day administration. (E) Food intake from 6th to 7th day of administration. (F) Body weight of mice before and after 7-day administration of PBS and ostruthin. Columns indicate the average body weights and each dotted line between columns indicates the weight change of each mouse. Both groups of mice insignificantly lost weight during the 7-day administration period. There were no differences between PBS- and ostruthin-treated mice at the end of the administration.

Nevertheless, a single administration showed antidepressive effects, but 7-day administration did not. To investigate the reason for this diminishment of effect, we next examined the blood concentration of ostruthin 30 min after the single- or 7-day administration. Ostruthin is a derivative of coumarin, which is known to be a good substrate for drug-metabolizing enzymes (Lake, 1999). Chronic administration may upregulate the hepatic drug-metabolizing enzymes thereby preventing the sufficient rise in drug concentrations and the manifestation of antidepressant effects. To test this possibility, blood samples were collected 30 min after the administration from both the single-shot and the 7-day regimen mice. After preparation and deproteinization of the serum, we analyzed ostruthin concentrations using a reverse-phase HPLC column and compared them (Fig. 5). The peak height of the serum prepared from a 7-day regimen was significantly lower than that from a single administration (Fig. 5B and C). The mean blood concentration was 8.14 ± 1.90 μM in single administration mice, whereas that of the 7-day regimen was 2.30 ± 0.95 μM (Fig. 5D). These concentrations were higher and lower, respectively, than the EC_50_ of ostruthin for TREK-1 activation (5.3 μM) (Joseph et al., 2018).

Ostruthin has been reported to have some adverse biological activities, such as mitochondrial uncoupling (Dadák et al., 1980) and antiproliferative (Joa et al., 2011). It is also possible that the 7-day regimen resulted in the illness, leading to the diminishment of ostruthin’s antidepressant effect. We analyzed food intake and body weight changes to monitor the adverse effect at least in part. The food consumption was insignificantly increased in the ostruthin-treated mice (Fig. 5E). Insignificant decreases in the body weight were observed in both PBS- and ostruthin-treated mice during the administration period (Fig. 5 F), most likely due to food and water deprivation for NSF. At the end of 7-day administration period, there was no significant difference in the body weights between the two groups, suggesting the lack of substantial effects.

## 4 Discussion

Previously, we demonstrated that a single administration of a TREK-1 channel activator, ostruthin, has antidepressive and anxiolytic effects in acute behavioral test models in mice that were not subjected to chronic stress (Joseph et al., 2018). Since major depression in humans is a chronic disorder and antidepressants are taken for a long period of time, it is essential to evaluate the effects of long-term administration of ostruthin in a chronically stressed mouse model, which is thought to be similar to the clinical condition in humans (Antoniuk et al., 2019). Therefore, we tested the effect of the long-term administration of ostruthin on chronically stressed mice in this study. Unexpectedly, this antidepressant effect was diminished after 7 days of ostruthin administration, including the anxiolytic effect. Since the blood concentration of ostruthin was significantly reduced in the 7-day regimen mice, this diminishment is probably due to upregulation of the hepatic drug-metabolizing enzymes. In contrast, a single dose of ostruthin showed an antidepressant effect. These findings indicate that TREK-1 channel activators still could offer a promising therapeutic approach for depression and highlight a need for stable compounds that are less susceptible to drug metabolism.

It is known that coumarin-related compounds are metabolized by drug-metabolizing enzymes, such as cytochrome (CYP) P-450 (Dawson et al., 1985). Coumarin-related compounds can induce the CYP450 enzymes (Kuhn et al., 1998), thereby increasing the metabolizing activity (Foroozesh et al., 2019, Peters et al., 1991). The 7-day administration likely induced the drug-metabolizing activity, leading to the diminishment of the antidepressive effect. Indeed, the blood ostruthin concentration of the 7-day regimen was lower than that of single-shot mice. This finding also suggests a requirement and usefulness for *in silico* drug metabolism and pharmacokinetics predicting databases, such as DruMAP (Kawashima et al., 2023). Predicting the induction of drug-metabolizing activity and a decrease in the drug concentration could reduce the need for animal experiments.

Since ostruthin has been reported to have some adverse biological effects (Joa et al., 2011, Dadák et al., 1980) it is also possible that the mice became ill during the 7-day treatment, leading to the diminishment of the antidepressant effect. However, the 7-day administration showed no effect on body weight or food intake. Indeed, the mean blood concentration of ostruthin was below the concentrations reported for the antiproliferative effect (IC_50_, 19 μM) (Joa et al., 2011) and anti-cancer activity (EC_50_ 67 μM) (Nguyen et al., 2017). These adverse effects seem to have a minor influence on the disappearance of the antidepressive effect.

Our previous study demonstrated that the suppression of neuronal activity in the lateral septum (LS) is involved in the antidepressant effect (Joseph et al., 2018). Namely, ostruthin suppressed neuronal activity in the LS without affecting neuronal activity in other nuclei, such as the dorsal raphe nucleus, hypothalamic paraventricular nucleus, and amygdala. The LS is reported to play a role in freezing behavior and relay stress-related information to other nuclei (Mongeau et al., 2003). TREK-1 channel was reported to be expressed in the LS (Talley et al., 2001). Therefore, it is likely that the suppression of the neuronal activity through the activation of the channel in the LS leads to the antidepressant effects. On the other hand, this study did not evaluate effects on female mice. Clinical and animal studies have shown that there are sex differences in susceptibility to chronic stress and in response to antidepressants (Kokras and Dalla, 2014, Wittchen et al., 2011). In particular, neural circuits involving the LS have been reported to be involved in the sex difference in anxiety- and depression-related behaviors (Bangasser and Cuarenta, 2021, Bredewold and Veenema, 2018). There is also a sex difference in the expression of drug-metabolizing enzymes (Waxman and Holloway, 2009). If a TREK-1 channel activator that is less susceptible to drug-metabolizing enzymes is found, its antidepressant effects should be examined in both male and female mice.

## 5 Conclusions

A single administration of ostruthin showed an antidepressive effect in chronically stressed mouse models, indicating that the TREK-1 channel activator could be a promising candidate for future antidepressants. However, the effect was not observed after 7 days of ostruthin administration, likely due to drug-metabolizing activity. These results suggest a need to develop a TREK-1 channel activator that is stable with drug-metabolizing enzymes.

## CRediT authorship contribution statement

**Masayoshi Okada:** Conceptualization, Data curation, Formal analysis, Funding acquisition, Investigation, Project administration, Supervision, Validation, Writing – original draft, Writing – review & editing. **Tran Thi Thu Thuy:** Investigation, Resources, Writing – original draft.

## Declaration of Competing Interest

The authors declare that they have no known competing financial interests or personal relationships that could have appeared to influence the work reported in this paper.
